# A Power Supply Technology for a Low-Power Online Monitoring Sensor Based on Electric Field Induction

**DOI:** 10.3390/s19092169

**Published:** 2019-05-10

**Authors:** Zong Li, Hongwei Mei, Liming Wang

**Affiliations:** Graduate School at Shenzhen, Tsinghua University, Shenzhen 518055, China; z-li16@mails.tsinghua.edu.cn (Z.L.); mei.hongwei@sz.tsinghua.edu.cn (H.M.)

**Keywords:** power supply technology, low power, online monitoring sensor, electric field induction

## Abstract

In order to provide a safe and stable power supply for low-power online monitoring sensors of transmission lines, a method of harvesting space electric field energy by using the impedance conversion characteristics of the transformer and the reactive compensation characteristics of the capacitor is proposed. The method effectively solves the key problem that the power of energy harvesting based on electric field induction is limited by the transformer excitation reactance and is difficult to upgrade. In this paper, the principle of power supply technology based on electric field induction is described in detail, and the influencing factors such as the wire erection mode, polar plate installation position, and capacitance compensation characteristics are simulated and tested. The test results show that when the power supply voltage is 50 kV, the stray capacitance is 14 pF, the compensation capacitance is 0.86 nF, and the load resistance is 1 kΩ, the energy-harvesting power is 340 mW. Finally, the power supply circuit of a drive-away-birds apparatus is designed, which shows that it can provide a stable and reliable power supply for online monitoring sensors.

## 1. Introduction

A stable and reliable power supply is an indispensable part of an online monitoring sensor for transmission lines. Currently, the online monitoring sensor on the tower is mainly powered by solar photovoltaic (PV) generation and batteries [1]. However, solar PV generation is influenced by environmental factors. Extreme weather (strong wind and rain) will seriously affect the power supply stability and, because the battery life is limited, it is necessary to replace batteries within a certain amount of time. Therefore, it is necessary to develop a stable way of energy harvesting to cooperate with solar energy for power supply.

At present, in addition to solar photovoltaic power generation, the main methods of energy harvesting are coil-based magnetic energy harvesting and electric field induction energy harvesting. The coil-based magnetic energy harvesting installs the energy-harvesting coil on high-voltage transmission lines (HTVL) and uses the principle of mutual inductance to harvest the magnetic field energy around the HTVL [2]. Since the coil is installed on the transmission line, it is difficult to transfer energy to the online monitoring device on the tower. Electric field induction energy harvesting is based on the stray capacitance between the metal plate and the conductor on the tower [3,4,5]. Its power supply is stable and can directly supply power to the online device on the tower. It is a potential power supply mode for online monitoring devices of overhead transmission lines. Therefore, scholars have carried out detailed research on it.

In [3,4,5], the cylindrical metal plate was installed on the power transmission line, and the stray capacitance between the cylinder and the earth was used for energy harvesting. The test proved that the power of 17.3 mW could be harvested on 60 kV transmission lines. On the basis of previous studies [6,7,8], some works [9,10,11] introduced a transformer to adjust the load equivalent impedance, which greatly improved the load power. On this basis, another study [12] changed the operation mode of the energy-harvesting power supply and installed the energy-harvesting plate on the tower, which could directly supply power to the online monitoring sensor on the tower. In these studies [9,10,11,12], the impedance conversion characteristics of the transformer were used to increase the power of the load. However, when the equivalent impedance of the load increases to the same order of magnitude as that of the excitation reactance of the transformer, the excitation reactance begins to shunt, reducing the power of the load.

On the basis of these findings, this paper proposes a method for harvesting space electric field energy by using the impedance conversion characteristics of the transformer and the reactive compensation characteristics of the capacitor, which solves the key problem that the power of energy harvesting based on electric field induction is limited by the transformer excitation reactance and is thus difficult to upgrade. Although the resulting power is still relatively low, it is greatly improved compared to previous studies and meets the basic power supply demand of low-power online monitoring sensors.

## 2. Materials and Methods

### 2.1. Principle

**Figure 1:**
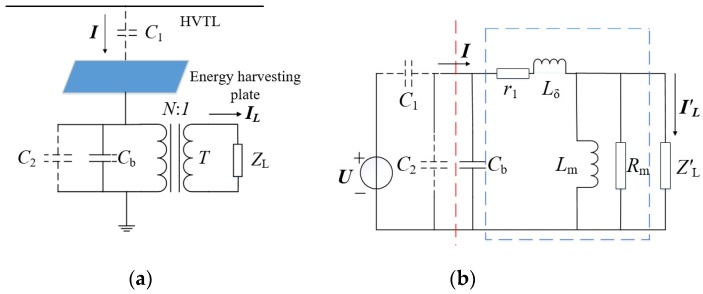
Proposed schematic and equivalent circuit model of electric field energy harvesting away from high-voltage transmission lines (HVTL). (**a**) Physical model and (**b**) equivalent circuit of the model.

Figure 1 shows the actual circuit diagram and the equivalent circuit model. As shown in Figure 1a, a metal plate was installed between the HTVL and the tower, and the metal plate was surrounded by the electric field generated by the HVTL. When the alternating-current (AC) electric field passed through the metal plate, an electric charge was induced on the surface of the metal plate, and the displacement current formed. The displacement current flew through the transformer and the compensation capacitor (*C*_b_), and finally transferred the energy to the load. 

The function of the transformer is to improve the equivalent impedance of the load to meet the requirements of the voltage and power of the load by using the impedance conversion characteristics. The function of *C*_b_ is to compensate for the inductive current of the inflow transformer and improve the power factor. In Figure 1a, *C*_1_ represents the stray capacitance between the HVTL and the metal plate, *C*_2_ is the stray capacitance between the metal plate and the ground, *N*:*1* represents the turn ratio of the transformer, and *Z*_L_ is the load impedance.

**Figure 2:**
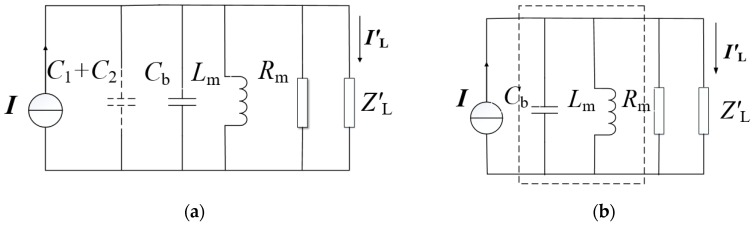
Simplification of the circuit model. (**a**) Norton equivalent circuit and (**b**) final simplified circuit.

Figure 1b shows the equivalent circuit model. *R*_m_ and *L*_m_ are the equivalent excitation resistance and excitation inductance, respectively; *Z*′_L_ is the equivalent load impedance; and *r*_1_ and *L*_δ_ are the wire-wound resistor and leakage inductance, respectively. In order to facilitate the analysis, the equivalent circuit was simplified. The parameters *r*_1_ and *L*_δ_ are generally much smaller than the excitation impedance; therefore, these two terms were neglected here. At the same time, the equivalent simplified circuit diagram in Figure 2a could be obtained by the Norton equivalent transformation of the left circuit of the red line. The equivalent constant current source is *I* = *ωC*_1_*U*. The equivalent capacitance is *C*_1_ + *C*_2_. Since *C*_1_ + *C*_2_ ≪ *C*_b_, *C*_1_ + *C*_2_ can be neglected, the final simplified circuit is obtained, as shown in Figure 2b.

From Figure 2b, it is easy to obtain the equivalent impedance of the dotted line frame as follows:

(1) $$Z_{S}=\frac{j\omega L_{m}}{1-\omega^{2}C_{{}_{b}}L_{m}}$$

Thus, the output current *I′_L_* can be obtained by

(2) $$I_{L}^{\prime }=I\left|\frac{R_{m}Z_{S}}{R_{m}Z_{S}+R_{m}Z_{L}^{\prime }+Z_{S}Z_{L}^{\prime }}\right|$$

The load power can be expressed as

(3) PL=IL′2Re(ZL′)

Formulae (1)–(3) show that the power is affected by stray capacitance, transformer characteristic parameters, compensation capacitance, and load impedance. In order to obtain the required power through parameter selection and impedance matching, the above factors were analyzed. 

### 2.2. Influence Factor

#### 2.2.1. Bundle Conductor

A bundle conductor is a kind of conductor erection method adopted by ultra-high voltage (EHV) transmission lines to restrain corona discharge and reduce line reactance. In order to study the stray capacitance of a double-bundle conductor and a single conductor, finite-element software was used to simulate the stray capacitance. 

**Figure 3:**
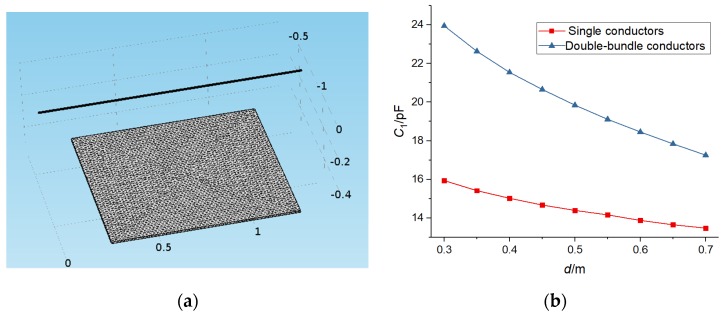
Simulation diagram and simulation results of the bundle conductors. (**a**) Simulation diagram and (**b**) *C*_1_ change curves of single- and double-bundle conductors.

Figure 3a shows that the single conductor was 1.5 m long and was located directly above the metal plate, the double-bundle conductor was 1.5 m long, the distance between the two conductors was 40 cm, and the center of the two conductors was located directly above the metal plate. The length of the plate was 1m, and the distance between the conductor and the plate was d = 0.3:0.1:0.6. The stray capacitance changed in two different ways, as shown in Figure 3b.

As can be seen from Figure 3b, the stray capacitance of double-bundle conductors at different distances was greater than that of single conductors. The double-bundle conductor could effectively increase the radius of the conductor, the vertical component of the electric field line through the plate, and the induced charge. According to the formula *i* = *dq*/*dt*, the space displacement current increased, that is, the stray capacitance increased. Therefore, under the same conditions, from the formulae (1)–(3), it can be seen that the device installed under the double-bundle conductors was able to obtain a greater power.

#### 2.2.2. Plate Installation Position

The influence of the stray capacitance between the plate and the three-phase conductor should be considered when selecting the installation position of the metal plate. 

**Figure 4:**
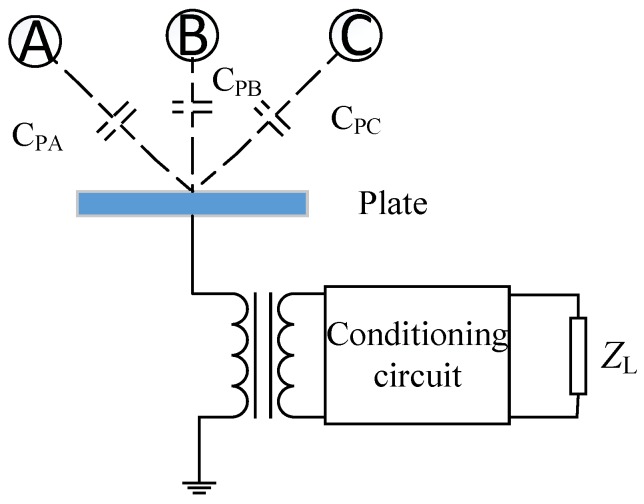
Power circuit model of a three-phase transmission line.

As shown in Figure 4, the equivalent constant current source is

*I* = *jω*(*U*_A_*C*_PA_ + *U*_B_*C*_PB_ + *U*_C_*C*_PC_) (4)

and three-phase AC transmission lines are characterized by

*U*_A_ + *U*_B_ + *U*_C_ = 0 (5)

Therefore, when selecting the installation location, *C*_PA_ = *C*_PB_ =*C*_PC_ should be avoided, otherwise the power gain will be zero. In order to avoid *C*_PA_ = *C*_PB_ =*C*_PC_ in engineering applications, the plate should be as close as possible to a phase conductor, so that the stray capacitance value between the plate and the phase is much higher than that of the other two phases, so as to improve the output power.

#### 2.2.3. Matching Principle of Transformer and Compensation Capacitor

Formulae (1)–(3) show that the larger the equivalent resistance *Z*_S_, the larger the load shunt, and the larger the load power. The size of *Z*_S_ is affected by *L*_m_, *C*_b_, and *ω*; since *ω* is a constant, only the excitation inductance *L*_m_ and the compensation capacitance *C*_b_ need to be analyzed. 

**Figure 5:**
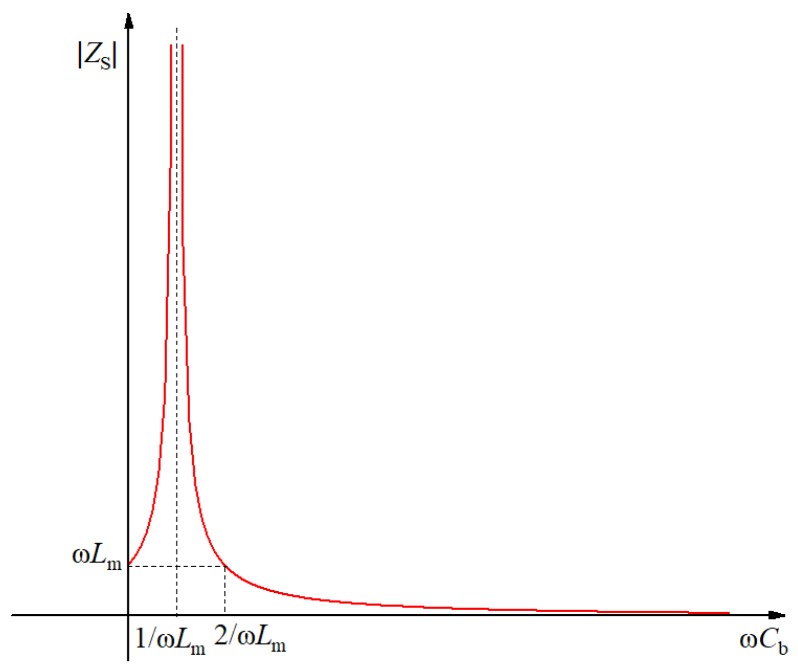
Change curve of *Z*_S_ with compensation capacitance.

Figure 5 shows the variation curve of the *Z*_S_ absolute value with compensation capacitance. When *C*_b_ is zero, *Z*_S_ is ω*L*_m_, i.e., an excitation reactance value; when 0 < ω*C*_b_ < 1/ω*L*_m_, *Z*_S_ gradually increases from *L*_m_ to +∞, so *Z*_S_ is still inductive and in the compensation stage; when ω*C*_b_ = 1/ω*L*_m_, parallel resonance occurs, and *Z*_S_ tends to +∞ at the full compensation stage; when 1/ω*L*_m_ < ω*C*_b_ < 2/ω*L*_m_, *Z*_S_ decreases gradually from +∞ to ω*L*_m_; when ω*C*_b_ > 2/ω*L*_m_, *Z*_S_ decreases gradually from ω*L*_m_ to 0.

From the analysis of Figure 5 and the above formulae, it can be seen that when ω*C*_b_ ≈ 1/ω*L*_m_, *Z*_S_ tends to +∞, and the current flowing through *Z*_S_ is almost zero; therefore, under the same conditions, the current flowing through load *Z*’_L_ is the largest, and the compensation capacitance *C*_b_ is about 1/ω^2^*L*_m_.

#### 2.2.4. Analysis of Excitation Impedance Parameters 

From the above analysis, when ω*C*_b_ = 1/ω*L*_m_, i.e., *Z*_S_ = ∞, the load is able to obtain the maximum power. In fact, the precision of the compensation capacitor and the influence of *C*_1_ and *C*_2_ make it impossible to obtain full compensation. Assuming that the error is 10% (the general capacitance accuracy is 10%), *C*_b_ can be obtained from Formula (1), and ω*C*_b_ = 1/ω*L*_m_.

(6) $$Z{}_{S}=\frac{j\omega L{}_{m}}{1-\omega^{2}L{}_{m}C{}_{b}\left(1\pm 10\%\right)}=\pm 10j\omega L{}_{m}$$

Formula (2) shows that *Zs* is not completely infinite and shunts with *Z*_m_ (*Z*’_L_//*R*_m_). Assuming that the load is a resistive load,

(7) $$P{}_{L}={\left|\frac{\left|\alpha Z{}_{m}\right|j}{\left|\alpha Z{}_{m}\right|j+\left|Z{}_{m}\right|}\right|}^{2}I^{2}\left|Z{}_{m}\right|=\left(\frac{1}{1+\frac{1}{\alpha^{2}}}\right)I^{2}\left|Z{}_{m}\right|$$

(8) $$\left|Z{}_{S}\right|=\alpha \left|Z{}_{m}\right|$$

If the energy-harvesting power is above 90% of the ideal state, then

(9) $$\frac{1}{1+\frac{1}{\alpha^{2}}}\geq 90\%\Rightarrow \alpha \geq 3$$

(10) $$\frac{10\omega L{}_{m}}{\left|Z{}_{m}\right|}\geq \alpha \Rightarrow \omega L{}_{m}\geq 0.3\left|Z{}_{m}\right|$$

According to Formula (10), the excitation inductance of the transformer should be designed according to the equivalent load impedance, and its minimum value should be greater than 0.3|*Z*_m_|.

#### 2.2.5. Load-Side Parameter Matching Analysis

Load-side parameters match needs to analyze the corresponding relationship between the turn ratio and the load impedance. Formulae (1)–(3) show that *P*_L_ = *I*_L_^2^ *Re*(*Z*_L_), and when parallel compensation occurs in the circuit, *I_L_* will remain unchanged, so the load power is only related to the equivalent impedance of the load. *Z′*_L_ = *N*^2^ *Z*_L_, so, according to different load impedance values, the transformer ratio *N* can be adjusted to get the same equivalent impedance to meet the load power requirements.

## 3. Results and Discussion

### 3.1. Experimental Analysis of Bundle Conductor

**Figure 6:**
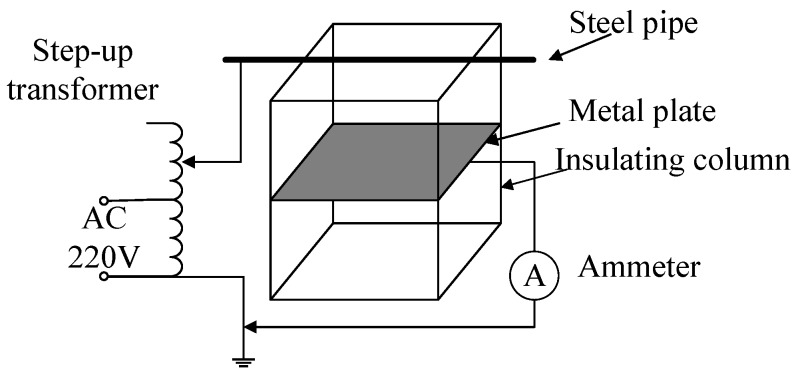
Displacement current measuring circuit.

**Figure 7:**
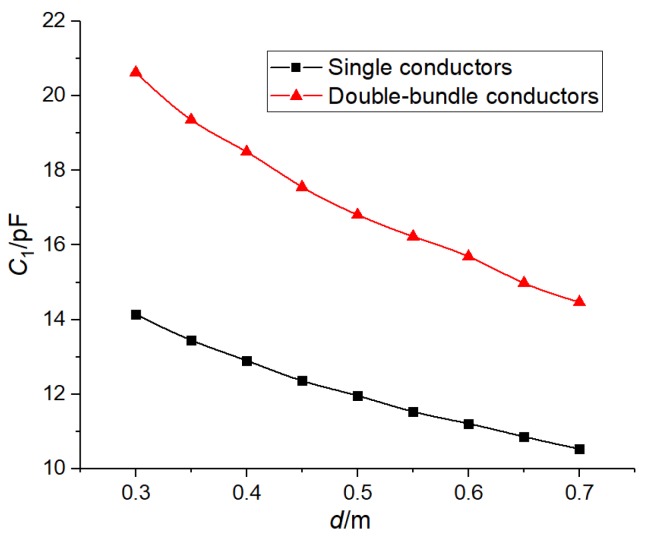
*C*_1_ change curves of single- and double bundle conductors.

In order to verify the influence of the bundle conductor on the stray capacitance, a displacement current measurement circuit, as shown in Figure 6, was built in the laboratory. The design of the test parameters was consistent with that of 2.2.1 simulation parameters. The test voltage was 50 kV, and the frequency was 50 Hz. The displacement current *I_1_* was measured by an ammeter. The stray capacitance values could be obtained from *I_1_* = *ωC_1_U*. Figure 7 shows the variation of the stray capacitance values with the distance in two erection modes.

Because of the test environment and the measuring instrument, the stray capacitance value measured by the test deviated from that obtained by the simulation. However, it is obvious that the stray capacitance of the double-bundle conductor was larger than that of the single-bundle conductor, and the measured curve was almost the same as the simulation curve.

### 3.2. Test and Analysis of the Energy-Harvesting Circuit

**Figure 8:**
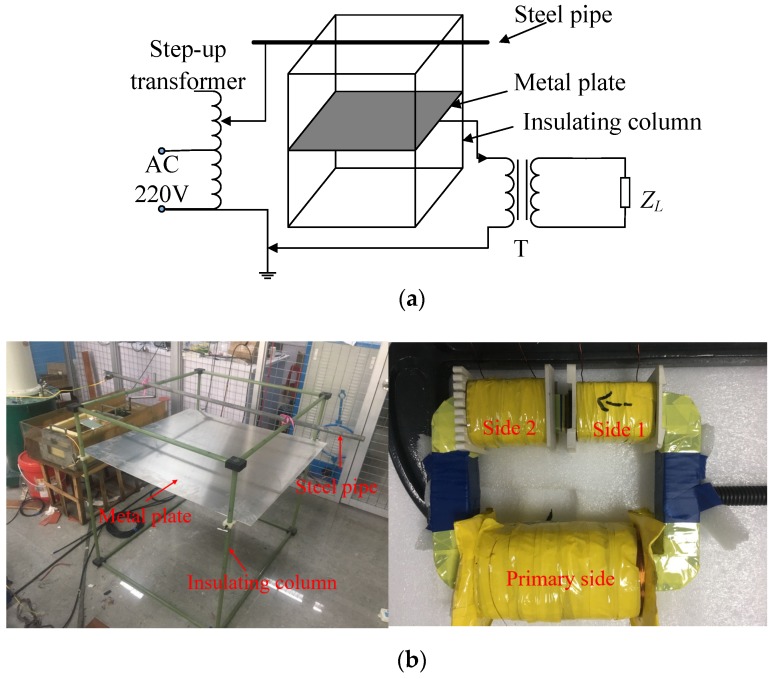
Test circuit. (**a**) Test schematic diagram and (**b**) physical photo.

In order to verify the validity of the electric field induction energy-harvesting technology, a test circuit was designed as shown in Figure 8a, and some of the test pictures are shown in Figure 8b.

The AC (220 V) was raised to 50 kV by a boost transformer and added to a 1.5 m-long steel pipe. The steel pipe was supported by four insulating pillars to ensure insulation between the steel pipe, metal plate, and earth. A square plate with a length of 1 m was installed parallel to the bottom of the steel tube at 0.3 m. The stray capacitance *C*_1_ between the steel tube and the plate was about 14 pF, and the stray capacitance *C*_2_ between the plate and the earth was about 35 pF. A transformer formed the primary side of the connection plate and ground, and the connection load formed the secondary side. An oscilloscope was used to measure the load voltage waveform.

#### 3.2.1. Load Characteristic Analysis of the Transformer

**Figure 9:**
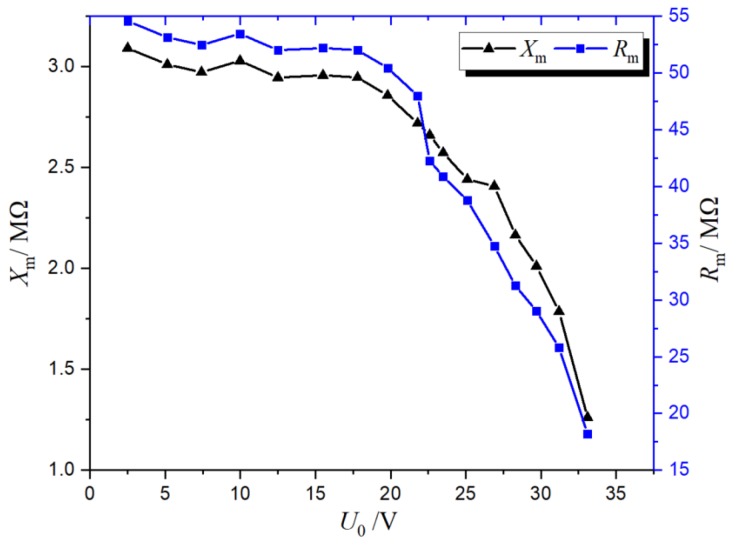
*R_m_* and *X_m_* variations with the load voltage.

An energy-harvesting transformer is the key device of electric field induction energy harvesting and needs to be tested and analyzed first. According to the analysis described in 2.2.4, the excitation impedance was greater than 0.3|*Z*_m_|, and the value of |*Z*_m_| was usually larger to obtain a larger power, so the value of excitation inductance was also very large. Therefore, in order to improve the excitation inductance, the core of the transformer was a Fe-based amorphous alloy, which presented a high saturation magnetic induction, a high initial permeability, and very low power loss. The primary winding of the transformer was 20,000 turns, side 1 was 200 turns, and side 2 was 400 turns. The curves of *R*_m_ and *X*_m_ varying with load voltage were obtained by applying the voltage to side 1, as shown in Figure 9.

It can be seen from Figure 9 that when the voltage was less than 20, the core was in the linear region of the magnetization curve, and the relative magnetic permeability was essentially unchanged, so the excitation reactance (3 MΩ) and the excitation impedance (50 MΩ) were also essentially unchanged. When the voltage was greater than 20 or less than 25, with the increase of voltage, the core tended to be saturated, the relative magnetic permeability began to decrease (from 2.8 MΩ to 2.5 MΩ), the excitation reactance began to decrease (from 50 MΩ to 38 MΩ), and the excitation resistance also began to decrease. When the voltage was greater than 25 V, the core began to saturate, the relative magnetic permeability decreased rapidly, the excitation reactance decreased rapidly, and the excitation current rapidly increased, causing the excitation resistance to drop rapidly.

**Figure 10:**
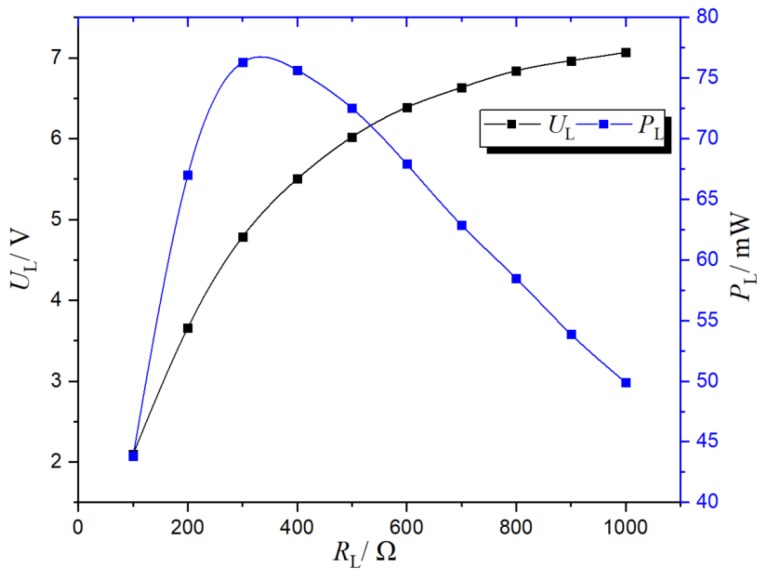
Load characteristics of the transformer.

Figure 10 shows the load characteristic curve of the transformer (the load was connected to the secondary side 1). Without adding the compensation capacitor, the load voltage increased linearly first and then tended to be stable with the increase of the load; the output power of the load increased first and then decreased with the increase of the load and reached a maximum value between 300 and 350.

#### 3.2.2. Influence of Compensation Capacitance and Excitation Impedance on Power

At the test voltage of 50 kV, the frequency was 50 Hz, the distance between the steel pipe and metal plate was 0.3 m, the load impedance was 1 kΩ, and the turn ratio was *N* = 100. The curve of representing changes of the load voltage with the compensation capacitance *C*_b_ was obtained.

**Figure 11:**
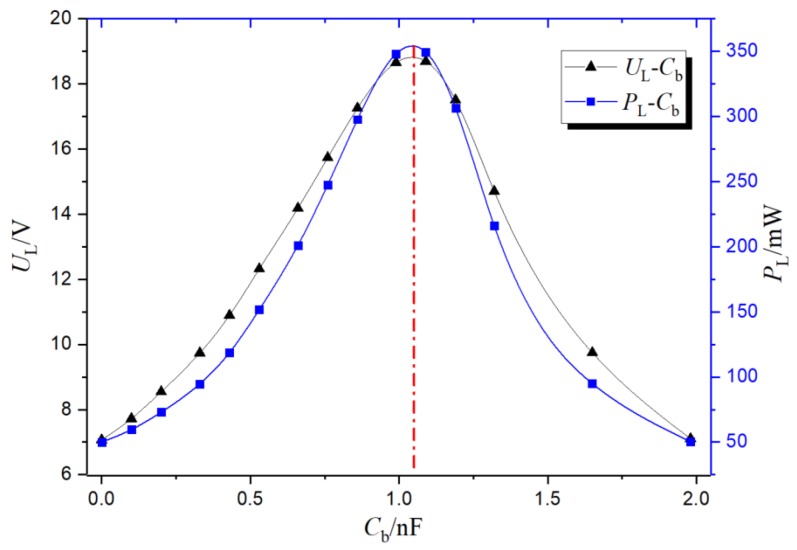
Compensation curve.

From the compensation curve shown in Figure 11, it can be seen that when *C*_b_ was in an under-compensation state (0 < ω*C*_b_ < 1/ω*L*_m_) at 0–1.04 nF, the inductive current was larger than the capacitive current, the sum of the inductive current and capacitive current flowing through *Z*_S_ was smaller and smaller, and the load voltage *U*_L_ and the energy-harvesting power *P*_L_ increased with the increase of *C*_b_.

When *C*_b_ = 1.04 nF (estimated value), a near full compensation state was reached (ω*C*_b_ = 1/ω*L*_m_). The sum of inductive current and capacitive current flowing through *Z*_S_ was almost zero. Load voltage *U*_L_ and energy-harvesting power *P*_L_ reached the maximum, where *U*_Lmax_ > 18.7 V, and *P*_Lmax_ > 349 mW (consistent with the theoretical calculation from Formula (11)).

(11) $$U_{\mathrm{Lmax}}=\frac{1}{N}\omega C_{1}U\frac{R_{L}^{\prime }}{R_{L}^{\prime }+R{}_{m}}R{}_{m}=18.5V$$

When *C*_b_ was in the over-compensated state (1/ω*L*_m_ < ω*C*_b_ < 2/ω*L*_m_) at 1.04 Nf–2 nF, the capacitive current value gradually began to be larger than the inductive current. The sum of the inductive current and capacitive current flowing through *Z*_S_ increased, and the load voltage *U*_L_ and the energy-harvesting power *P*_L_ gradually decreasd with the increase of *C*_b_.

From the above experimental analysis, it can be seen that the fitting curve of the measured data basically conformed to the theoretical analysis in Section 2.2.3.

#### 3.2.3. Parameter matching between Turn Ratio and Load

**Figure 12:**
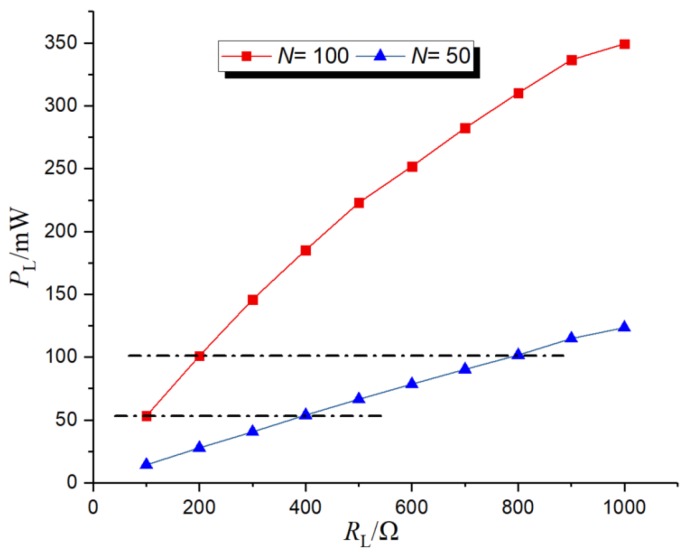
The relation between the turn ratio and the impedance of the load.

At the test voltage of 50 kV, the frequency was 50 Hz, the distance between the steel pipe and metal plate was 0.3 m, *C_b_* = 0.86 nF, and the turn ratio was 100 and 50, respectively. The curve of load power versus load resistance is shown in Figure 12. 

As can be seen from Figure 12, the load power increased linearly with the increase of load resistance at the same turn ratio in the case of parallel resonance, which was consistent with the theoretical analysis. The power of the same load at the turn ratio N = 100 was greater than that at the turn ratio *N* = 50. When parallel resonance occurred, according to *Z*´_L_ = *N^2^Z*_L_, the bigger the turn ratio, the bigger the equivalent impedance, and the bigger the load power. At the same time, it was found that the load power obtained by the turn ratio *N* = 100, *R*_L_ = 100 Ω was basically equal to that obtained by the turn ratio *N* = 50, *R*_L_ = 400 Ω, because the equivalent impedance of the two was equal, so the load power was the same, which was consistent with the analysis in Section 2.2.5.

### 3.3. Application of Electric Field Energy Source

In order to verify the effectiveness of the energy-harvesting method based on electric field induction, the power supply circuit of a drive-away-birds apparatus for transmission line towers was designed. The specific parameters are shown in Table 1.

According to the parameters, the effective working range of the apparatus was 80–100 m^2^, which met the requirements to protect the tower from birds. Under the working condition, its power was 140 mW, and its equivalent impedance was 280 Ω. In a standby state, the power was 5 mW, and the equivalent impedance was 7.75 kΩ.

**Figure 13:**
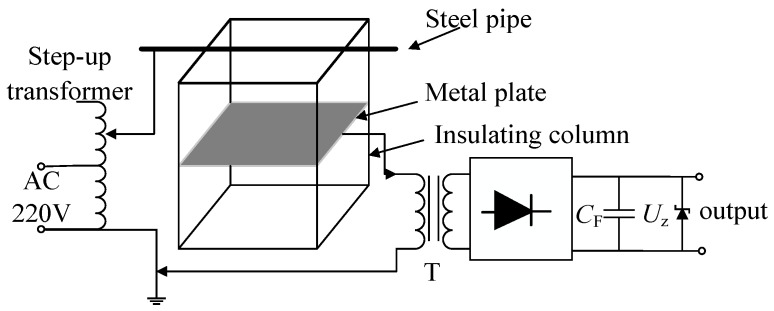
The power supply circuit.

According to the power requirement of the drive-away-birds apparatus, the power supply circuit was designed as shown in Figure 13.

From the above analysis, it is known that when the load impedance increases, *U*_L_ and *P*_L_ will also increase, which is opposite to the power requirement of the drive-away-birds apparatus. When in its working state, the equivalent resistance of the apparatus is small, and the power requirement is large; in a standby state, the equivalent resistance is large, and the power requirement is small. Therefore, we could use a low-power Zener diode to keep its working voltage stable at 6.2 V. When the apparatus was in a working state, the Zener diode did not work, while, when it was in a standby state, the equivalent impedance increased, and the voltage rose. At this time, the Zener diode started to work and maintained the voltage at 6.2 V.

**Figure 14:**
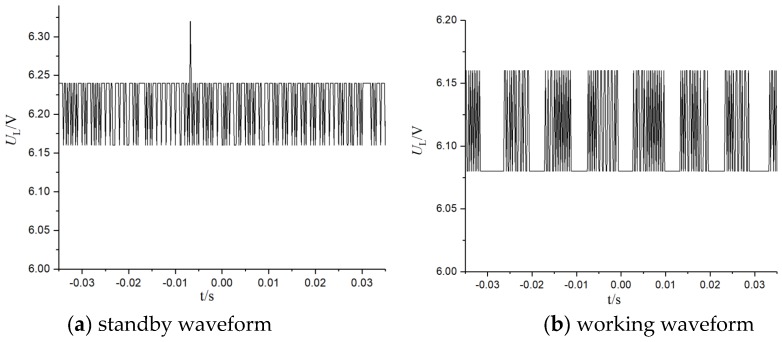
Test waveform of the drive-away-birds apparatus.

Because of its lower equivalent impedance under working conditions, the energy was smaller. In order to obtain enough power, the number of secondary side turns was adjusted to 120 turns (*Z′*_L_ = 6 MΩ). In order to obtain a smoother waveform, it was necessary that *C*_F_ = 2200 uF. The test record waveform is shown in Figure 14.

From the test results, the load voltage was basically stable at 6.2 V in both working and standby conditions, and the drive-away-birds apparatus could work normally and reliably, which basically verified the effectiveness of the energy-harvesting method based on electric field induction.

## 4. Conclusions

Currently, the harvesting efficiency of electric field energy harvesting by electric field induction is low and cannot meet the demands of online monitoring sensor power supplies. Considering this, a method of harvesting space electric field energy by using transformer impedance conversion characteristics and capacitor reactive power compensation characteristics is proposed in this paper. The method effectively solves the key problem that the power of energy harvesting based on electric field induction is limited by the transformer excitation reactance and is difficult to upgrade. Although the resulting power is still relatively low, it is greatly improved compared to previous studies and can basically meet the power supply demand of low-power online monitoring sensors. In this paper, through the analysis of the principle of the method and experimental analyses, it is proved that our method can effectively improve the efficiency of electric field energy harvesting. Finally, the power supply circuit of a drive-away-birds apparatus is presented, showing that it can provide a stable and reliable power supply for online monitoring sensors. 

## Figures and Tables

**Table 1:** Drive-away-birds apparatus parameters.

Working Voltage /V	Working Current /mA	Standby Current/mA	Effective Range/m^2^
6.2	22	0.8	80–100
